# Potential prognostic value of heat-shock protein 90 in the presence of phosphatidylinositol-3-kinase overexpression or loss of PTEN, in invasive breast cancers

**DOI:** 10.1186/bcr2557

**Published:** 2010-03-12

**Authors:** Chang Hoon Song, So Yeon Park, Keun-Yong Eom, Jee Hyun Kim, Sung-Won Kim, Jae Sung Kim, In Ah Kim

**Affiliations:** 1 Department of Radiation Oncology, Seoul National University Bundang Hospital, 300 Gumi-dong, Bundang-gu, Seongnam-si, Kyeongi-do, 463-707, Korea; 2 Department of Pathology, Seoul National University College of Medicine, Seongnam-si, Kyeongi-do, 463-707, Korea; 3 Breast Care Center, Seoul National University Bundang Hospital, 300 Gumi-dong, Bundang-gu, Seongnam-si, Kyeongi-do, 463-707, Korea

## Abstract

**Introduction:**

Evaluating the expression of signaling molecule proteins from the mitogen-activated protein kinase (MAPK) pathway and the phosphatidylinositol-3-kinase (PI3K) pathway in invasive breast cancers may identify prognostic marker(s) associated with early relapse.

**Methods:**

Immunohistochemical analyses of phosphatase and tensin homologue deleted on chromosome 10 (PTEN), PI3K-p110α, phospho-AKT, phospho-p70S6 kinase, phospho-S6 ribosomal protein, phospho-RAF, phospho-p44/42 MAPK, and heat-shock protein 90 (HSP90) were performed on tumor samples from 212 patients with invasive breast cancer. Statistically significant relations between protein expression, clinicopathologic factors, and relapse-free survival (RFS) were analyzed.

**Results:**

Expression of HSP90 was associated with 5-year RFS, as well as T stage, N stage, histologic grade, estrogen receptor (ER) expression, human epidermal growth factor receptor 2 (HER2) expression, and the Ki-67 proliferation index. On multivariate analysis, coexpression of HSP90 and PI3K-p110α or expression of HSP90 along with PTEN loss demonstrated significantly worse RFS. In subgroup analyses, both exhibited strong prognostic significance in HER2-positive cases, but not in HER2-negative cases.

**Conclusions:**

The coexpression of HSP90 with PI3K-p110α or expression of HSP90 along with PTEN loss has a potential as a molecular prognostic marker to predict early relapse in patients with invasive breast cancers.

## Introduction

Disruption of critical cell-signaling pathways responsible for cell growth and survival has been shown to contribute to atypical cell proliferation, to metastatic competence of breast cancer cells, and may be responsible for therapeutic resistance of some breast cancers. In the field of targeted breast cancer therapy, several prognostic markers including clinical stage [1], histologic grade [2], estrogen receptor (ER)/progesterone receptor (PR) status [3-5], human epidermal growth factor receptor-2 (HER2) [6,7], and the Ki-67 proliferation index [8] have already been identified and validated. Additional molecular markers that have potential prognostic significance also have been identified.

HER2 is a cell-surface receptor tyrosine kinase (RTK), which is amplified in one fourth of breast cancers and confers a more aggressive clinical course and worse prognosis [6,7]. The intracellular catalytic domain of HER2 receptor can be phosphorylated by the formation of homodimeric or heterodimeric kinase-active complexes, and it ultimately leads to activation of signal-transduction pathways that promote proliferation or survival of breast cancer cells: the mitogen-activated protein kinase (MAPK) and the phosphatidylinositol-3-kinase (PI3K) pathways are two major signal-transduction pathways of the ErbB family RTKs involved in proliferation and survival [reviewed in [9]]. AKT, p70 S6 kinase, and ribosomal protein S6 are among the most important and representative downstream molecules of these signaling pathways [10]. Another important protein of the PI3K pathway is the phosphatase and tensin homologue deleted on chromosome 10 (PTEN) [11]. PTEN antagonizes PI3K function and negatively regulates AKT activity. Conversely, heat-shock protein 90 (HSP90) is a chaperone required for the stability and function of these proteins, especially ER, HER2, AKT, and RAF [12]. Previous studies evaluated the prognostic significance of these molecular markers. Several of these proteins have shown potential as prognostic markers; however, these studies have examined each molecule individually [13-20].

In the present study, we analyzed the expression of target molecules involved in the HER2-associated signaling pathway including PTEN, p110α subunit of PI3K (PI3K-p110α), phosphorylated AKT (p-AKT), phosphorylated p70 S6 kinase (p-p70S6K), phosphorylated ribosomal protein S6 (p-S6), phosphorylated RAF (p-RAF), phosphorylated p44/42 MAPK (p-p44/42 MAPK), and HSP90. Additionally, the prognostic significance of these molecular markers, individually, as well as in combination with other established prognostic factors, was investigated.

Expression of HSP90 from invasive breast cancer was associated with an increased risk of early recurrence. Coexpression of HSP90 and PI3K or expression of HSP90 in combination with the loss of PTEN was associated with significantly worse RFS. In subgroup analysis, coexpression of HSP90 and PI3K-p110α or expression of HSP90 along with PTEN loss showed strong prognostic significance in patients with HER2-positive cancers, but not with HER2-negative cancer.

Thus, these results show that the coexpression of HSP90 with PI3K-p110α or the expression of HSP90 along with PTEN loss has a potential as a molecular prognostic marker for early relapse in patients with invasive breast cancers.

## Materials and methods

### Patients and samples

Tissue samples from 212 patients who underwent surgical resection for primary invasive breast cancer at Seoul National University Bundang Hospital from May 2003 to November 2005 were collected. Informed consent for pathologic evaluation was obtained from patients from whom tissue samples were taken. After surgery, most patients were treated with the standard practice guidelines and have been followed up regularly.

Paraffin-embedded tumor samples from the 212 patients with invasive breast cancer were obtained in accordance with a protocol approved by the institutional review board of Seoul National University Bundang Hospital. The following histopathologic variables were determined by reviewing pathology reports and hematoxylin and eosin-stained sections: tumor size, lymph node metastasis, histologic subtype, and histologic grade. Tissue microarrays were constructed for a high-throughput study as previously described [21], as well as for immunohistochemical staining.

### Immunohistochemical analysis

After staining optimization by using positive and negative controls, eight antibodies that provided satisfactory results, together with standard prognostic biomarkers, including ER, PR, HER2, and Ki-67, were used for the study (Additional file 1). Immunohistochemical staining was carried out by using the DAKO Envision detection kit (Dako, Carpinteria, CA). In brief, tissue array blocks were cut into 4 μm-thick sections, dried, deparaffinized, and rehydrated. Antigen retrieval was performed in a microwave oven for 15 min in 10 m*M *citrate buffer, pH 6.0, and endogenous peroxidase activity was blocked with a 3% H_2_O_2_-methanol solution. The slides were blocked with 10% normal goat serum for 10 minutes and were incubated with an appropriately diluted primary antibody for an hour at room temperature or overnight at 4°C. The slides were then probed with HRP-labeled polymer conjugated to secondary antibody for 30 minutes. Diaminobenzidine was used as a chromogen, and the sections were counterstained with Mayer's hematoxylin.

### Definition of breast tumor subtypes

Breast tumor subtypes were defined as follows: luminal A (ER^+ ^and/or PR^+^, HER2^-^), luminal B (ER^+ ^and/or PR^+^, HER2^+^), HER2^+^/ER^- ^(ER^-^, PR^-^, HER2^+^), and triple negative (ER^-^, PR^-^, HER2^-^). HER2 positivity was determined based on FISH results from the previous study [21].

### Interpretation and statistical analysis

Staining results were interpreted by a breast pathologist who was blinded to patient outcomes. The expression of ER, PR, and Ki-67 was scored as positive if >10% nuclear staining was found. Immunohistochemical scoring of signaling molecules was based on a semiquantitative method according to the percentage of positive cells and the intensity of staining. Staining in either the cytoplasmic or the nuclear compartment was all considered positive (Additional file 1). The percentage of positive cells was scored as follows: 0, no staining or staining in <5% of the tumor cells; 1, staining in 5% to 25% of the cells; 2, staining in 26% to 50% of the cells; 3, staining in 51% to 75% of the cells; and 4, staining in >75% of the cells. The staining intensity was scored as 0 (negative), 1 (weak), 2 (moderate), or 3 (strong). For all signaling molecules except PTEN, cases with >5% of positive tumor cells with moderate to strong intensity were grouped as positive. For PTEN, cases with complete loss of staining were grouped as negative, and cases with any staining, as positive.

All data were analyzed with SPSS 12.0 for Windows (SPSS Inc., Chicago, IL). The Spearman correlation test was used to analyze the association between variables. Relapse-free survival (RFS) was measured from the date of diagnosis to the date of locoregional recurrence, distant metastasis, or death. Kaplan-Meier survival curves for RFS were constructed, and differences were determined by log-rank test. To test the independent prognostic significance of certain markers, each marker was included with the standard prognostic factors into a Cox proportional hazards model.

## Results

Patients and tumor characteristics are presented in Table 1. At the time of the analysis, the median follow-up was 5 years (range, 3 to 6 years). There were seven (3%) locoregional recurrences as first events and 19 (9%) distant metastases. Eleven patients had died of recurrent or metastatic breast cancer.

**Table 1:** Patients and tumor characteristics

Characteristics	Number of patients (%)	
Age (years)		
<40	33	(15.6)
≥40	179	(84.4)
Histology		
Invasive ductal	205	(96.7)
Invasive lobular	7	(3.3)
Tumor stage		
T1--T2	199	(93.9)
T3--T4	13	(6.1)
Nodal stage		
N0	106	(50.0)
N1--N3	106	(50.0)
Histologic grade		
Grade I--II	116	(54.7)
Grade III	89	(42.0)
ER		
Negative	70	(33.0)
Positive	142	(67.0)
PR		
Negative	113	(53.3)
Positive	99	(46.7)
HER2		
Negative	168	(79.2)
Positive	44	(20.8)
Ki-67 index		
<10%	120	(56.6)
≥10%	91	(42.9)

ER = estrogen receptor; HER2 = human epidermal growth factor receptor-2; PR = progesterone receptor.

**Figure 1:**
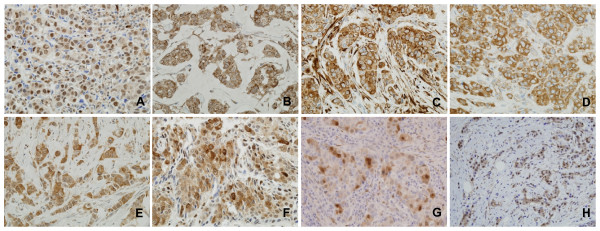
**Immunohistochemical analyses of various signaling molecules in MAPK and PI3K pathways**. Representative examples of immunohistochemical staining patterns observed in invasive breast cancers. p-AKT (a) shows predominant nuclear staining with weak cytoplasmic staining in this case. PI3K-p110α (b), p-S6 (c), p-RAF (d), and HSP90 (e) show strong cytoplasmic staining. The p-p70S6K (f), p-p44/42 MAPK (g), and PTEN (h) are localized in both cytoplasmic and nuclear compartments.

PTEN loss was found in 26.9%; PI3K-p110α was expressed in 66.3%; p-AKT, in 28.3%; p-p70S6K, in 75.8%; p-S6, in 62.7%; p-RAF, in 45.7%; p-p44/42 MAPK, in 26.8%; and HSP90, in 63.5% of the 212 tissue samples of invasive breast cancers (Table 2; Figure 1).

**Table 2:** Expression rates of the signaling proteins tested

Marker	Number of positive cases/Total number of cases (%)	
PTEN loss	57/212	(26.9)
PI3K-p110α	138/208	(66.3)
p-Akt	60/212	(28.3)
p-p70S6K	160/211	(75.8)
p-S6	131/209	(62.7)
p-RAF	96/210	(45.7)
p-MAPK	56/209	(26.8)
HSP90	134/211	(63.5)

HSP90 = heat-shock protein 90; MAPK = mitogen-activated protein kinase; PI3K = phosphatidylinositol-3-kinase; PTEN = phosphatase and tensin homologue deleted on chromosome 10.

Table 3 summarizes the correlations between the markers analyzed and the histopathologic features of the tumors. Expression of p-AKT was associated with high histologic grade (*P *= 0.006) and HER2 amplification (*P *< 0.001). Expression of p-p70S6K was correlated with high histologic grade (*P *= 0.035), ER negativity (*P *< 0.001), and HER2 amplification (*P *< 0.001). A significant correlation was seen between p-S6 and high histologic grade (*P *< 0.001), ER negativity (*P *< 0.001), HER2 amplification (*P *= 0.004), and high Ki-67 proliferation index (*P *< 0.001). The expression of p-AKT, p-p70S6K, and p-S6, which are involved in the PI3K pathway, demonstrated a significant positive correlation with each other. Expression of p-RAF and p-p44/42 MAPK, which are constituents of the MAPK pathway, also exhibited a positive correlation (*P *< 0.001). HSP90 expression showed a significant positive correlation with p-RAF expression (*P *= 0.047). Although not statistically significant, HSP90 expression demonstrated a trend toward correlation with the presence of p-AKT (*P *= 0.062), but not with ER or HER2 (*P *= 0.178 and *P *= 0.471, respectively).

**Table 3:** Correlation matrix of histopathologic variables and expression of each signaling protein

	≥ T3	LN+	Grade 3	ER	HER2	Ki-67	PTEN	PI3K-p110α	p-Akt	p-p70S6K	p-S6	p-Raf	p-p44/42-MAPK
≥ T3	-												
LN+	**0.010**	-											
Grade 3	**0.044**	0.185	-										
ER	0.100	0.772	** < 0.001**	-									
HER2	0.361	**0.042**	** < 0.001**	** < 0.001**	-								
Ki-67	0.821	0.236	** < 0.001**	** < 0.001**	** < 0.001**	-							
PTEN	0.746	0.644	**0.015**	**0.042**	0.753	**0.031**	-						
PI3K-p110α	0.151	0.770	0.236	0.159	0.085	0.768	0.552	-					
p-Akt	0.668	0.762	**0.006**	0.702	** < 0.001**	0.060	0.284	0.211	-				
p-p70S6K	0.925	0.139	**0.035**	** < 0.001**	** < 0.001**	0.057	0.780	0.192	**0.002**	-			
p-S6	0.930	0.606	** < 0.001**	** < 0.001**	**0.004**	** < 0.001**	0.172	0.553	**0.011**	** < 0.001**	-		
p-Raf	0.974	0.671	0.509	0.770	0.328	0.800	0.343	0.266	** < 0.001**	** < 0.001**	0.364	-	
p-p44/42 MAPK	0.756	0.562	0.238	0.623	0.841	0.118	0.428	0.334	0.147	**0.015**	0.974	** < 0.001**	-
HSP90	0.302	0.510	0.588	0.178	0.471	0.759	0.481	0.065	0.062	0.153	0.208	**0.047**	0.278

Variables found to be significant (*P *< 0.05) are shown in bold. ER = estrogen receptor; HER2 = human epidermal growth factor receptor-2; HSP90 = heat-shock protein 90; MAPK = mitogen-activated protein kinase; PI3K = phosphatidylinositol-3-kinase; PTEN = phosphatase and tensin homologue deleted on chromosome 10.

**Figure 2:**
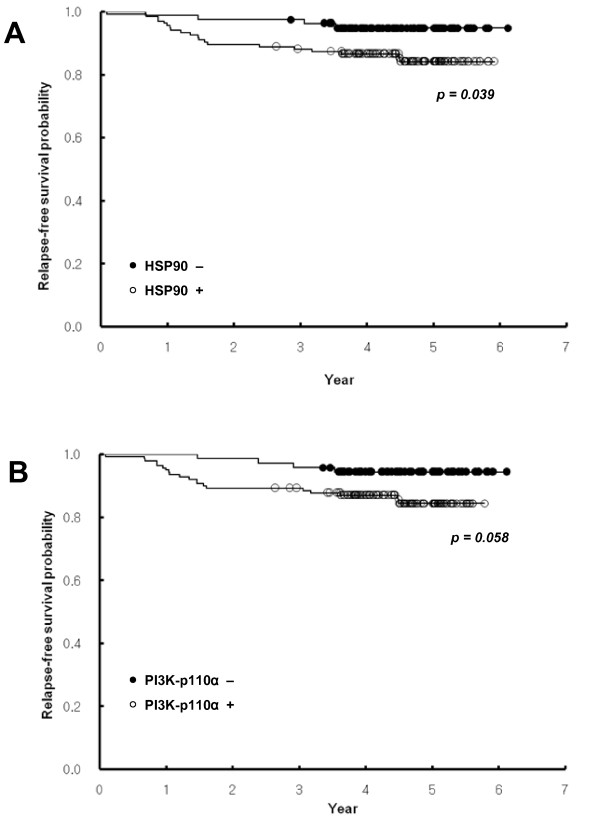
**Kaplan-Meier survival curves showing the probability of relapse-free survival for patients with invasive breast cancer in relation to the expression of HSP90 (a) and PI3K-p110α (b)**.

In univariate analyses, several conventional prognostic factors were identified as significant predictive factors for RFS, including tumor stage, nodal stage, histologic grade, and amplification of HER2. ER had only marginal significance for a correlation with RFS (*P *= 0.052). Of the other signaling molecules analyzed, HSP90 was significantly associated with RFS (*P *= 0.039; Figure 2A). PI3K-p110α had a trend toward poor RFS (*P *= 0.058; Figure 2B). PTEN, p-AKT, p-p70S6K, p-S6, p-RAF, and p-p44/42 MAPK were not associated with RFS (Table 4).

**Table 4:** Predictive factors for relapse-free survival*

Variable	*P *value
T stage	** < 0.001**
N stage	** < 0.001**
Histologic grade	**0.033**
ER	0.052
PR	0.076
HER2	**0.016**
Ki-67	**0.015**
PTEN	0.397
PI3K-p110α	0.058
p-Akt	0.681
p-p70S6K	0.342
p-S6	0.504
p-Raf	0.729
p-p44/42 MAPK	0.366
HSP90	**0.039**

*Based on univariate analysis. Variables found to be significant (*P *< 0.05) are shown in bold.ER = estrogen receptor; HER2 = human epidermal growth factor receptor-2; HSP90 = heat-shock protein 90; MAPK = mitogen-activated protein kinase; PI3K = phosphatidylinositol-3-kinase; PR = progesterone receptor; PTEN = phosphatase and tensin homologue deleted on chromosome 10.

**Figure 3:**
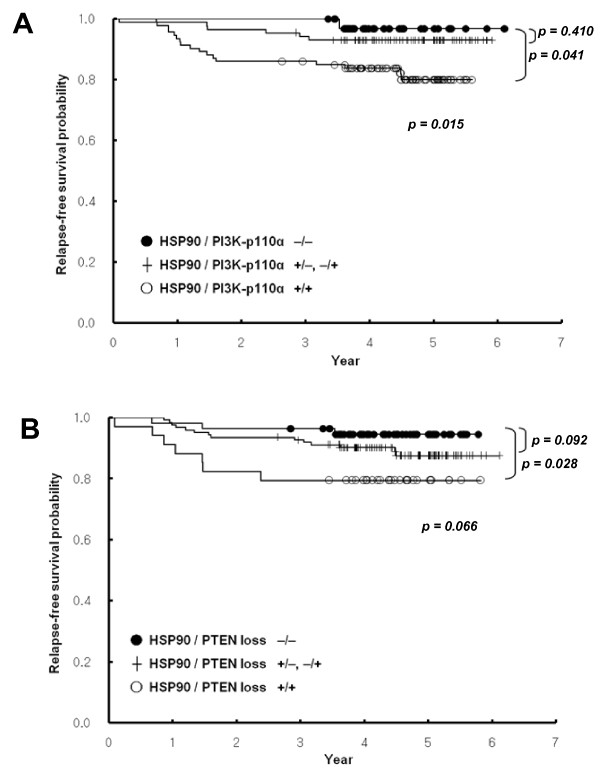
**Kaplan-Meier survival curves showing the probability of relapse-free survival for patients with invasive breast cancer in relation to the expression status of HSP90 and either PI3K-p110α (a) or PTEN loss (b)**.

HSP90 did not remain significantly correlated to RFS (*P *= 0.269) in a multivariate analysis accounting for standard factors such as T stage (T1-2 versus T3-4), N stage (N0 versus N1-3), histologic grade (Grade 1-2 versus 3), ER, and HER2. However, when the expressions of both HSP90 and PI3K-p110α were included in the Cox proportional hazards model, the coexpression of HSP90 and PI3K-p110α was identified as an independent prognostic factor for RFS (Hazard ratio (HR), 2.99; 95% confidence interval (CI), 1.18 to 7.60; *P *= 0.021), as did T stage and N stage. The combination of HSP90 expression and PTEN loss was also a significant predictor for RFS in multivariate analysis (HR, 2.83; 95% CI, 1.11 to 7.20; *P *= 0.029). RFS curves plotted in relation to the expression status of HSP90 and either PI3K-p110α or PTEN are shown in Figure 3A and 3B, respectively. Other combined expression of HSP90 with p-AKT or p-p70S6K or p-S6 or p-RAF or p-p44/42 MAPK failed to show prognostic significance in multivariate analysis.

**Figure 4:**
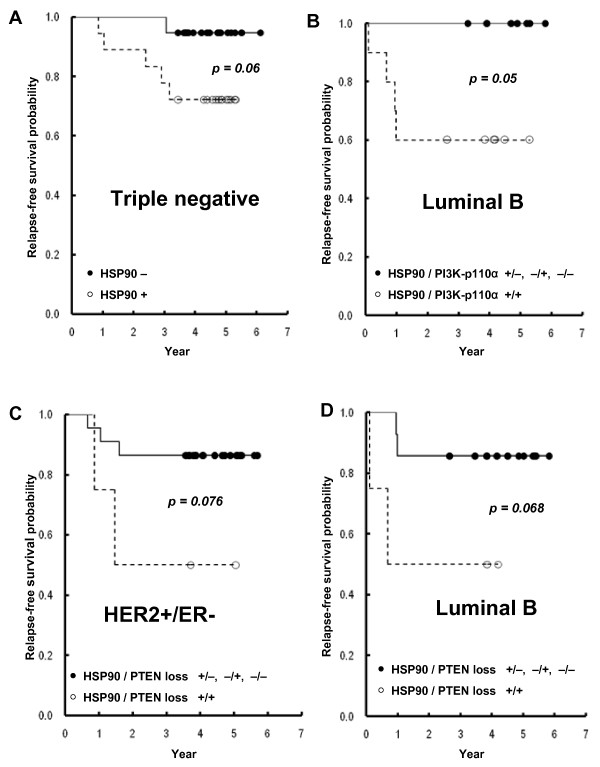
**Kaplan-Meier survival curves showing the probability of relapse-free survival for patients with invasive breast cancer in relation to the expression of HSP90 in triple-negative subtype (a), coexpression of HSP90 and PI3K-p110α in luminal B subtype (b), expression of HSP90 along with PTEN loss in HER2+/ER-subtype (c) and in luminal B subtype (d)**.

When the tumors were classified according to intrinsic subtypes, 131 (62%) cases were classified as luminal A, 18 (9%) cases as luminal B, 26 (13%) cases as HER2+/ER-, and 37 (18%) cases as triple-negative subtype. Table 5 shows expression rates of HSP90, PI3K-p110α, and PTEN loss within each intrinsic subtype. Triple-negative cases exhibited a significantly higher rate of loss of PTEN compared with those of other subtypes. The prognostic value of HSP90, PI3K-p110α, and PTEN was also evaluated according to the individual intrinsic subtype. Although not statistically significant, HSP90 expression demonstrated a trend toward correlation with worse RFS in the triple-negative subtype (Figure 4A). Coexpression of HSP90 and PI3K-p110α was a significant prognostic factor for RFS in luminal B subtype (Figure 4B). HSP90 expression, along with loss of PTEN, also showed a marginal prognostic significance in HER2^+^/ER^- ^and luminal B subtypes, respectively (Figure 4C and 4D).

**Table 5:** Expression of HSP90, PI3K-p110α, and PTEN loss according to intrinsic subtypes

	Luminal A (*n* = 131)		Luminal B (*n* = 18)		Triple negative (*n* = 37)		HER2+/ER- (*n* = 26)		*P *value
					
	*n*	(%)	*n*	(%)	*n*	(%)	*n*	(%)	
HSP90	86	(66)	13	(72)	18	(49)	17	(65)	0.208
PI3K-p110α	79	(62)	13	(72)	25	(68)	21	(81)	0.296
PTEN loss	29	(22)	6	(33)	17	(46)	5	(19)	0.023

HSP90 = heat-shock protein 90; PI3K = phosphatidylinositol-3-kinase; PTEN = phosphatase and tensin homologue deleted on chromosome 10.

**Figure 5:**
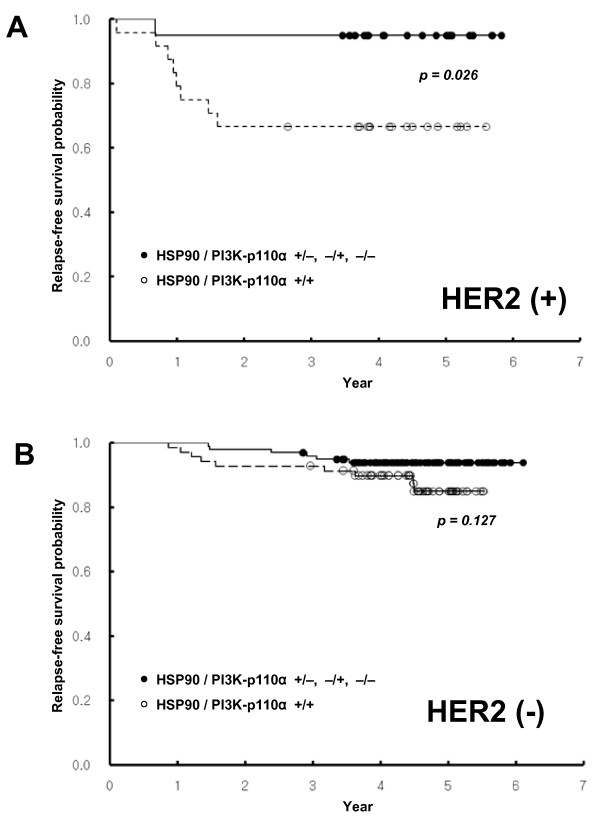
**Kaplan-Meier survival curves showing the probability of relapse-free survival for patients with invasive breast cancer in relation to the coexpression of HSP90 and PI3K-p110α in patients who are HER2 positive (a) or negative (b)**.

**Figure 6:**
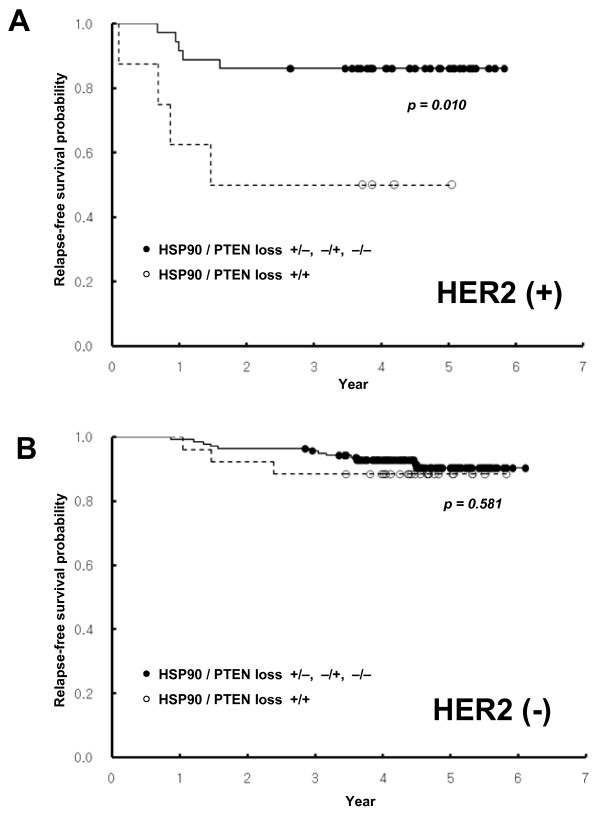
**Kaplan-Meier survival curves showing the probability of relapse-free survival for patients with invasive breast cancer in relation to HSP90 expression along with PTEN loss in patients who are HER2 positive (a) or negative (b)**.

When tumors were clustered by HER2 status, coexpression of HSP90 and PI3K-p110α predicted significantly worse RFS in patients with HER2-positive cases that included luminal B and HER2^+^/ER^- ^subtypes, whereas it failed to show prognostic significance in HER2^- ^cases (Figure 5). The HSP90 expression along with loss of PTEN also exhibited a strong prognostic significance in patients with HER2-positive cases, but not with HER2-negative cases (Figure 6).

## Discussion

In recent years, improvements in the understanding of the altered molecular events leading to breast cancer have led to the identification of new molecular targets and the development of new targeted therapies. Identification of the prognostic significance of HER2 and its targeted therapy provides the best examples of proof of concept [22]. Other potential negative prognostic factors represent a new opportunity for therapeutic targeting to improve treatment outcomes [23]. Growing evidence supports the dependence of cancer cells on aberrant PI3K signaling for survival. PI3K generates specific inositol lipids (PIP2 and PIP3) that have been implicated in the regulation of cell proliferation, differentiation, survival, and angiogenesis [reviewed in [24,25]]. Recently, several groups reported that 18% to 40% of human breast cancers harbor somatic mutations of the p110α catalytic subunit of PI3K [26], resulting in constitutive activation of PI3K signaling. The PTEN gene dephosphorylates PIP3, the critical lipid second messenger of PI3K. The loss of PTEN leads to accumulation of PIP3 and increases PI3K-AKT signaling [27]. Berns and colleagues [28] reported that activation of the PI3K pathway through loss of the tumor suppressor PTEN or through oncogenic stimulation of PI3K can mediate trastuzumab resistance. The current study also suggests that a potential prognostic significance of PI3K signaling may predict early relapse in patients with invasive breast cancers.

We previously reported that inhibitors targeting the PI3K-AKT pathway enhanced radiation-induced cell killing in human cancer cells [29]. We also have shown that targeting the PI3K-AKT-mTOR pathway can be an alternative strategy to overcome therapeutically resistant breast cancer cells with an activated HER2 signaling pathway. Impairment of DNA-damage repair increased apoptosis and autophagy and was identified as a mechanistic elements of increased breast cancer cell death [30].

The majority of breast cancers involve multiple molecular abnormalities that are likely to be involved in malignant progression. It is possible that several different molecules from diverse pathways have synergistic properties that promote malignant relapse or metastasis. In that situation, HSP90 could be a pivotal key molecule, as its chaperone function ensures the correct conformation, activity, intracellular localization, and proteolytic turnover of a range of proteins involved in cell growth, differentiation, and survival [12]. This molecular chaperon is essential for the stability and function of many oncogenic client proteins, which contributes to the hallmark trait of cancers such as ER, HER2, and AKT [31]. The inhibition of multiple targets through the abrogation of HSP90 could be more effective in the management of breast cancers, because its inhibition counteracts multiple oncogenic molecules and prosurvival signaling pathways at the same time.

Additional data support the identification of HSP90 as an important molecular target relevant to breast cancers. HER2, which is associated with poor prognosis in breast cancer, is one of the most important client proteins of HSP90, and HSP90 inhibitors have shown antitumor activity in an HER2-driven xenograft model [32]. Additionally, HSP90 inhibitors have been known to bind selectively to HSP90 in cancerous cells versus normal cells [33]. Breast cancer cells resistant to conventional chemotherapy, radiation therapy, and trastuzumab are known to involve the PI3K signaling pathway. The key molecule of this pathway, AKT, is also an important client protein of HSP90. A recent report suggests that the expression of HSP90 may be associated with poor clinical outcomes in patients with breast cancers [20]. The current study provides direct evidence that the expression of HSP90 predicts early relapse in patients with invasive breast cancers and validates the significance of HSP90 as a clinically significant therapeutic target.

Breast cancer is a heterogeneous entity, and the prognostic importance of intrinsic subtype has been extensively studied by gene-expression profiling. Almost all tumors having an intrinsic subtype of basal-like, HER2^+ ^and ER^-^, or luminal B were also classified as having a poor 70-gene profile and high recurrence score [34]. The largest meta-analysis of gene-expression profiles of 2,833 breast tumors also revealed connections between traditional prognostic factors, expression-based subtyping, and prognostic signatures [35]. In accord with the recent report by Marty and associates [36], triple-negative cases showed significantly higher rates of loss of PTEN. The exploratory analyses regarding intrinsic subtype were focused on the prognostic value of HSP90, PI3K-p110α, and PTEN in each intrinsic subtype; HSP90 expression exhibited a trend toward worse prognosis in patients having a triple-negative subtype. Coexpression of HSP90 and PI3K-p110α was a significant prognostic factor in patients having luminal B subtype. When combining the cases having luminal B (HER2^+^/ER^+^) and HER2^+^/ER^- ^subtypes, coexpression of HSP90 and PI3K-p110α or expression of HSP90 along with PTEN loss demonstrated strong prognostic significance in terms of RFS in patients with HER2-positive cancers, but not with HER2-negative cancer.

## Conclusions

Taken together, our data demonstrate the potential prognostic value of the coexpression of HSP90 and PI3K or the expression of HSP90 along with loss of PTEN. This also serves to refine further the characteristics of breast cancer patient subgroups with poor prognoses that could develop into early relapse. Therapeutic strategies that target these molecules may also be reasonable approaches to improving therapeutic outcomes for patients with invasive breast cancers.

## Abbreviations

ER: estrogen receptor expression; HER2: human epidermal growth factor receptor-2; HSP90: heat-shock protein 90; MAPK: mitogen-activated protein kinase; PI3K: phosphatidylinositol-3-kinase; PTEN: phosphatase and tensin homologue deleted on chromosome 10; RFS: relapse-free survival.

## Competing interests

The authors declare that they have no competing interests.

## Authors' contributions

IAK designed this study and is responsible for the preparation of the manuscript. SYP contributed to the pathologic work. CHS and KYE contributed to the management of clinical data. SWK, JHK, JSK, IAK provided expertise in clinical breast oncology.

## Supplementary Material

Additional file 1 **Table S1**. Antibodies used in this study. Click here for file
