# Supplementary material for: Potential prognostic value of heat-shock protein 90 in the presence of phosphatidylinositol-3-kinase overexpression or loss of PTEN, in invasive breast cancers
Source: Breast Cancer Res. 2010 Mar 12;12(2):R20. doi: 10.1186/bcr2557 (PMC2879564; doi:10.1186/bcr2557)
Supplement: Additional file 1 — Table S1. Antibodies used in this study. [file bcr2557-S1.PPT]

## Slide 1
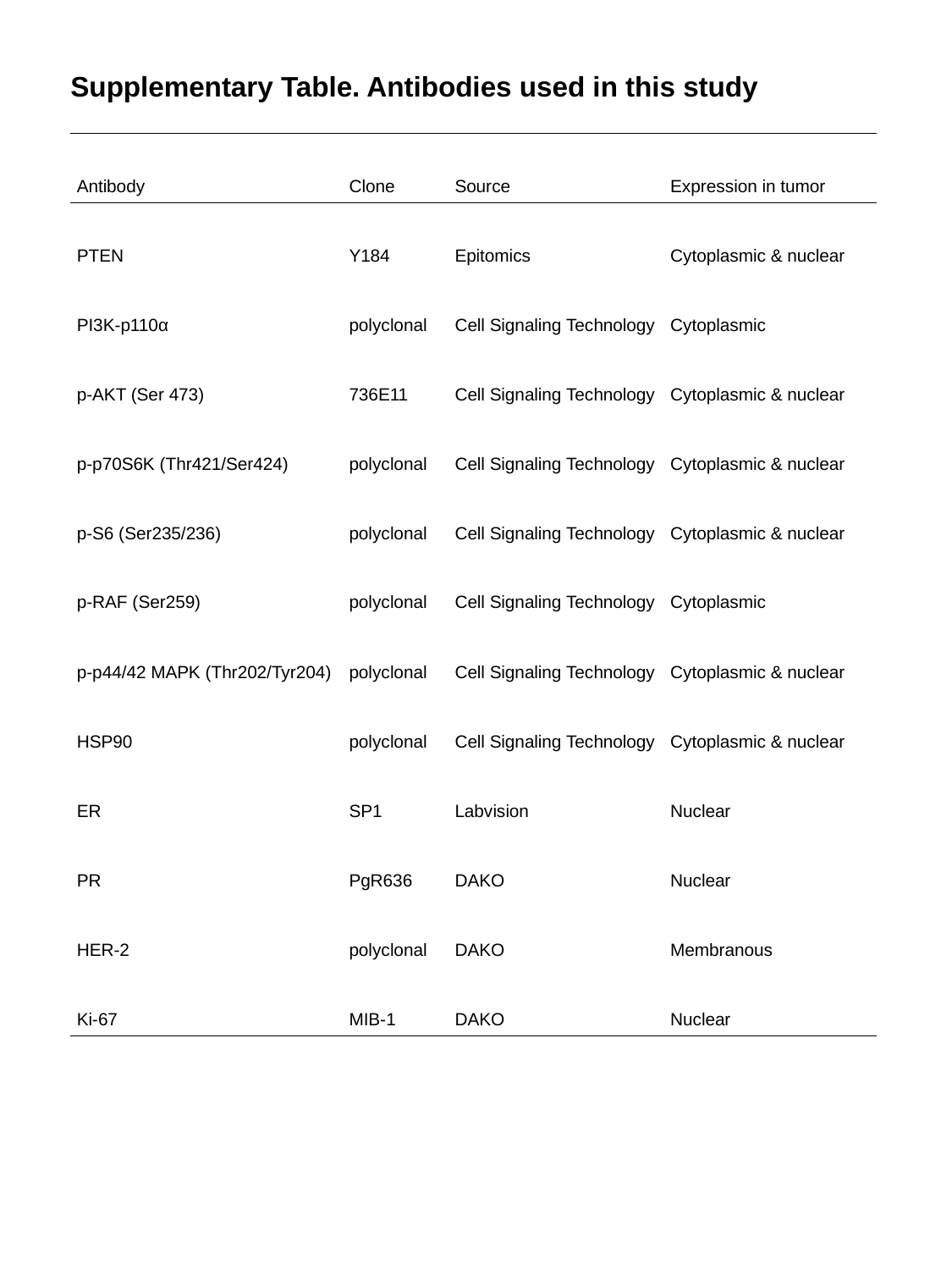

Supplementary Table. Antibodies used in this study
| Antibody | Clone | Source | Expression in tumor |
| --- | --- | --- | --- |
| PTEN | Y184 | Epitomics | Cytoplasmic & nuclear |
| PI3K-p110α | polyclonal | Cell Signaling Technology | Cytoplasmic |
| p-AKT (Ser 473) | 736E11 | Cell Signaling Technology | Cytoplasmic & nuclear |
| p-p70S6K (Thr421/Ser424) | polyclonal | Cell Signaling Technology | Cytoplasmic & nuclear |
| p-S6 (Ser235/236) | polyclonal | Cell Signaling Technology | Cytoplasmic & nuclear |
| p-RAF (Ser259) | polyclonal | Cell Signaling Technology | Cytoplasmic |
| p-p44/42 MAPK (Thr202/Tyr204) | polyclonal | Cell Signaling Technology | Cytoplasmic & nuclear |
| HSP90 | polyclonal | Cell Signaling Technology | Cytoplasmic & nuclear |
| ER | SP1 | Labvision | Nuclear |
| PR | PgR636 | DAKO | Nuclear |
| HER-2 | polyclonal | DAKO | Membranous |
| Ki-67 | MIB-1 | DAKO | Nuclear |
